# *Theileria equi* isolates vary in *s*usceptibility to imidocarb dipropionate but demonstrate uniform *in vitro* susceptibility to a bumped kinase inhibitor

**DOI:** 10.1186/s13071-014-0611-6

**Published:** 2015-01-20

**Authors:** Siddra A Hines, Joshua D Ramsay, Lowell S Kappmeyer, Audrey OT Lau, Kayode K Ojo, Wesley C Van Voorhis, Donald P Knowles, Robert H Mealey

**Affiliations:** Department of Veterinary Microbiology and Pathology, College of Veterinary Medicine, Washington State University, Pullman, WA 99164-7040 USA; Animal Disease Research Unit, Agricultural Research Service, USDA, Pullman, WA 99164-6630 USA; Division of Allergy and Infectious Diseases and Center for Emerging and Re-emerging Infectious Diseases, School of Medicine, University of Washington, Seattle, WA 98109-4766 USA

**Keywords:** *Theileria equi*, Equine piroplasmosis, Imidocarb dipropionate, Apicomplexan, Bumped kinase inhibitor, Drug susceptibility

## Abstract

**Background:**

The apicomplexan hemoparasite *Theileria equi* is a causative agent of equine piroplasmosis, eradicated from the United States in 1988. However, recent outbreaks have sparked renewed interest in treatment options for infected horses. Imidocarb dipropionate is the current drug of choice, however variation in clinical response to therapy has been observed.

**Methods:**

We quantified the *in vitro* susceptibility of two *T. equi* isolates and a lab generated variant to both imidocarb dipropionate and a bumped kinase inhibitor compound 1294. We also evaluated the capacity of *in vitro* imidocarb dipropionate exposure to decrease susceptibility to that drug. The efficacy of imidocarb dipropionate for clearing infection in four *T. equi* infected ponies was also assessed.

**Results:**

We observed an almost four-fold difference in imidocarb dipropionate susceptibility between two distinct isolates of *T. equi*. Four ponies infected with the less susceptible USDA Florida strain failed to clear the parasite despite two rounds of treatment. Importantly, a further 15-fold decrease in susceptibility was produced in this strain by continuous *in vitro* imidocarb dipropionate exposure. Despite a demonstrated difference in imidocarb dipropionate susceptibility, there was no difference in the susceptibility of two *T. equi* isolates to bumped kinase inhibitor 1294.

**Conclusions:**

The observed variation in imidocarb dipropionate susceptibility, further reduction in susceptibility caused by drug exposure *in vitro*, and failure to clear *T. equi* infection *in vivo*, raises concern for the emergence of drug resistance in clinical cases undergoing treatment. Bumped kinase inhibitors may be effective as alternative drugs for the treatment of resistant *T. equi* parasites.

## Background

*Theileria equi*, one of the two causative agents of equine piroplasmosis, is a tick-transmitted hemoprotozoan parasite classified within the phylum Apicomplexa. The United States has been considered free of equine piroplasmosis for several decades, however the occurrence of multiple U.S. outbreaks in recent years has caused a resurgence of interest in effective treatment options. Infection can be clinical or subclinical, but persists even with resolution of clinical signs [1-3]. Strict federal regulations for the elimination of infected horses have been established in an attempt to maintain piroplasmosis-free status in the U.S., requiring *T. equi* positive horses to be euthanized, permanently quarantined, exported to the country of origin, or treated under the current USDA-ARS-APHIS treatment program [4,5]. This program is currently the only federally-sanctioned option for treatment in the U.S., as full elimination of parasites from the host must be verified in order for treated horses to no longer be considered potential reservoirs of infection [5].

For most apicomplexan parasitic pathogens, the goal of treatment is to minimize the clinical impact of disease. Complete elimination of this type of pathogen is a considerable challenge, particularly with organisms such as *T. equi* which causes persistent infection [3]. Imidocarb dipropionate (IMD) is a dicationic diamidine of the carbanilide series of antiprotozoal compounds, and is the drug most commonly used to treat equine piroplasmosis caused by both *T. equi* and *Babesia caballi*. In a recent major outbreak localized in Texas [6], IMD successfully cleared over 163 naturally infected horses of *T. equi* (Dr. Angela Pelzel, USDA-APHIS, personal communication) [4,5]. However, variation in response to treatment with an identical IMD protocol has been observed in both natural and experimental *T. equi* infection [4,7-10], with treatment failure characterized by parasite persistence and recrudescence of parasitemia following discontinuation of treatment. The identification of drug resistance in other apicomplexan parasites [11-13] indicates drug resistance is likely an important factor in *T. equi* treatment failures. In particular, the human malarial agent *Plasmodium falciparum* has exhibited continuously evolving multidrug resistance, necessitating continued development of novel antimalarial drugs for effective treatment. Importantly, failure of treatment with previously effective drug protocols is almost invariably associated with decreased *in vitro* susceptibility to the treatment drug [11,14].

Many drugs have been assessed *in vitro* for efficacy against *T. equi* [15-21]; however a large number of these are not biologically relevant or feasible for use in horses. Although IMD is commonly used clinically, susceptibility has never been evaluated *in vitro* for *T. equi* nor compared between parasite strains. Importantly, the potential impact of IMD exposure on the susceptibility of *T. equi* to this drug, a known factor in the development of drug resistance in many other organisms [14,22-27], has not been investigated.

Given the scarcity of treatment options for *T. equi* and the potential for drug resistance, evaluation of alternative and novel drugs is necessary. Bumped kinase inhibitors (BKIs) are a group of experimental compounds currently being investigated for *in vitro* and *in vivo* efficacy against malaria [28,29], toxoplasmosis [30,31], cryptosporidiosis [31,32], and other protozoal diseases [33]. The BKIs selectively inhibit apicomplexan calcium-dependent protein kinases (CDPKs), which are critical for multiple parasitic physiological functions including parasite motility and invasion as well as in secretory pathways and replication [28]. Importantly, these CDPKs are absent in vertebrates, making them excellent anti-apicomplexan chemotherapeutic candidates [34]. Specifically, BKIs are competitive inhibitors of ATP-binding, and gatekeeper residue size appears to be a major factor in the selectivity of BKIs. These residues in apicomplexan CDPKs are small, typically glycine, serine, or threonine [28,34], which allow access to the ATP-binding pocket for BKIs to bind and inhibit apicomplexan CDPKs. Although CDPKs are not present in mammals, binding of most other mammalian kinases by BKIs is prevented by gatekeeper amino acid residues with large side chains that occlude access to the ATP-binding pocket. Therefore, the BKIs do not inhibit the proliferation of mammalian cells, and have been shown to be non-toxic in rodents [28,29,32].

In the present study, we evaluated the *in vitro* growth inhibitory effects of IMD against two isolates of *T. equi*, as well as a variant that was exposed *in vitro* to the drug. We also describe four ponies infected experimentally that failed to clear *T. equi* despite two rounds of IMD treatment following the established protocol (4 mg/kg, IM, q72 hrs for four doses) [7]. We then evaluated the *in vitro* efficacy of a novel bumped kinase inhibitor, BKI compound 1294, against two *T. equi* isolates with different degrees of susceptibility to IMD. This BKI compound was equally effective against both *T. equi* isolates, including the variant exposed *in vitro* to IMD. The results of this work should have implications in the design of therapeutic strategies against infections caused by drug-resistant *T. equi*.

## Methods

### Chemical reagents

For HL2A-FBS and –NHS culture media, HL-1 and HEPES were obtained from Fisher Scientific (Waltham, MA), HB101 supplement from Irvine Scientific (Santa Ana, CA), L-glutamine and AlbuMax from Gibco (Grand Island, NY) and penicillin/streptomycin and gentamicin from Sigma-Aldrich (St. Louis, MO). Hydroethidine was acquired from Invitrogen (Carlsbad, CA) as a 5 mM solution solubilized in DMSO. Imidocarb dipropionate (Imizol®, Merck, Millsboro, DE) at 344 mM (120 mg/mL) was utilized as the stock solution for all IMD assays and diluted in medium to reach experimental concentrations each day for use in the parasite growth inhibition assay described below. Bumped kinase inhibitor compound 1294 was generated as described [35], dissolved in DMSO at 20 mM, and then diluted in medium as above for use in the BKI 1294 growth inhibition assays.

### Evaluation of Theileria equi CDPK sequence

Relevant amino acid sequences of BKI-binding CDPK proteins from other apicomplexans [33] including *P. falciparum* (PfCDPK1 [GI:124801388]), *Babesia bovis* (BbCDPK4 [GI: 154796736]), and *T. gondii* (TgCDPK1 [GI:255917998]) were obtained from GenBank and BLASTed (blastp, NCBI) against amino acid sequences predicted in the *T. equi* genome (GenBank accession number: ACOU00000000) in order to identify the *T. equi* ortholog. These sequences were then aligned and analyzed using ClustalW and BoxShade programs.

### In vitro cultivation of Theileria equi

The USDA Florida strain of *T. equi* (FL) [36,37] was obtained as a subculture from ongoing USDA research cultures. Cultures were initially grown in a microaerophilic environment (5% O_2_) in modified HL2A-FBS medium [38] with 10 mM hypoxanthine, 200 U/mL penicillin, and 200 μg/mL streptomycin added. Over time, the cultures were adapted to ambient O_2_ in a 5% CO_2_ 37°C incubator and medium was converted from modified HL2A-FBS to modified HL2A-NHS (substituting normal horse serum for fetal bovine serum). Cultures were maintained in 24 well plates with an erythrocyte concentration of 10% in 1300 μL total well volume and split 1:4 every other day, with 1000 μL of medium changed on intervening days.

A novel *T. equi* isolate (TX) was obtained from a parasitemic horse as a part of a separate previous study [4] and adapted into culture. Briefly, an EDTA-anticoagulated whole blood sample was collected and centrifuged at 800 *g* for 30 minutes. The plasma and leukocytes were discarded, and the erythrocytes washed three times in an equivalent volume of VYMs buffer, centrifuging as before. These infected erythrocytes were then placed in one well of a 24 well plate with 1000 μL of media at a 1:1 ratio with uninfected normal horse erythrocytes (60 μL of each). The plate was initially placed in a 37°C incubator with 5% O_2_, 5% CO_2_, and 90% N_2_ gas, however following successful culture initiation, TX was subsequently adapted to ambient O_2_ in a standard 5% CO_2_ incubator and maintained as for the FL strain.

### *In vitro* parasite growth inhibition assay

Initial IMD concentrations to be used in the assay were determined based on the pharmacokinetics of the drug in horses [39] and the *in vitro* susceptibility of other organisms to the drug [22,40,41]. To evaluate the dynamics of parasite growth during IMD exposure and to determine the appropriate incubation time for the assay, the FL strain was cultured in a 24-well plate for 72 hours with assessment of parasite growth via flow cytometry (below) and one mL medium change every 24 hours. Serial dilutions of IMD were tested in triplicate ranging from 1 nM to 775 nM, with a target starting percent parasitized erythrocytes (PPE) for all wells of 0.25-0.5%.

Based on these results, it was determined that the assay should be performed over 72 hours, starting at a low initial PPE. Further assays were performed in a 96-well plate, with a final erythrocyte concentration of 9% and target starting PPE of approximately 0.3%. Two-fold serial dilutions of IMD ranged from 2.7 nM to 344 nM, and triplicate samples for each concentration, including infected untreated controls, were evaluated. Triplicate wells of normal uninfected erythrocytes were also included as a control comparison. For assay initiation, 2x drug concentrations were prepared, with 100 μL of the appropriate concentration placed into each well with 100 μL of normal medium and 20 μL of RBCs to reach the final 1× drug concentration for the assay and approximately 9% RBC concentration in each well. At 24 and 48 hours, 160 μL of medium containing the appropriate 1× drug concentration was changed in each well, with plain medium used for controls. The assay was then evaluated at 72 hours. Complete growth inhibition was considered to be achieved when the PPE remained at the starting level of 0.3-0.5% after 72 hours, indicating no active parasite growth.

The chemical structure of BKI 1294 has been previously published [30]. Drug concentrations tested for BKI 1294 included 0.3 μM, 3 μM, 6 μM, 9 μM, 12 μM, and 30 μM. Assay procedure was otherwise identical to that for IMD.

### Flow cytometric evaluation of parasite growth

After 72 hours, all samples were transferred to a 96 well V-bottom plate to be stained with hydroethidine. Cells were centrifuged at 500 *g* at room temperature, and the culture medium supernatant discarded. They were then washed in 150 μL of 1× PBS per well and centrifuged as before. Stock 5mM hydroethidine in anhydrous DMSO was diluted to 20 μg/mL in 1× PBS and added at to all samples (including normal erythrocytes) at 100 μL per well. Samples were incubated for 15 minutes at 37°C in 5% CO_2_ in the absence of light, and 100 μL of plain 1× PBS was added at the conclusion of the incubation prior to centrifugation. After removal of the supernatant, the cells were resuspended in 200 μL of 1× PBS. Fifty μL of each sample was then diluted into approximately two mL of 1× PBS containing 0.2% sodium azide for flow cytometric analysis.

Cell suspensions were evaluated using a FACSCaliber flow cytometer equipped with CellQuest computer software (Becton Dickinson Immunocytometry Systems, San Jose, CA) on a Macintosh computer. The inclusion gate was based on the forward and side scatter features of uninfected erythrocytes stained with hydroethidine, and 50,000-150,000 events per sample were collected. Ethidium bromide fluoresces in the FL-2 channel, therefore argon-laser fluorescence excitation at 488 nm and emission at 585 nm (range 563–607 nm) were used for analysis in log Fl 2 data mode. Fluorescent profiles were recorded for later analysis with FCS Express software (De Novo software, Los Angeles, CA). Quadrant gating of generated dot plots (Fl-2 vs. side scatter) was based on stained uninfected erythrocyte controls to delineate between infected and uninfected cell populations.

### Determination of IC_50_ and IC_90_ values

The PPE of each well was determined based on the percentage of the cell population characterized as RBCs that exhibited FL-2 fluorescence. Mean PPE for each drug concentration and controls was calculated by averaging the PPE of all three triplicate wells. Individual PPE values that deviated from the other replicates by >0.5% were considered outliers and eliminated from data evaluation. Percent of maximum PPE was then calculated for each concentration using the mean PPE value in comparison to the highest PPE obtained in the assay for a given isolate. The 50% (IC_50_) and 90% (IC_90_) inhibitory concentrations were determined by fitting the curves with nonlinear regression using GraphPad Prism version 6.03 for Windows (GraphPad Software, La Jolla, CA).

### Exposure of Theileria equi parasites to imidocarb dipropionate *in vitro*

Exposure of the FL strain of *T. equi* to IMD was performed via two different methods. The first method involved pulse exposure, with parasites exposed at 28.7 nM (the approximate IC_50_ value determined previously) for an initial period of 11 days, until growth had declined to a PPE of less than 1.5%. Drug pressure was then removed and the parasites were allowed to recover for five days. They were then re-exposed to 28.7 nM for 24 hours. Subsequent 24 hour re-exposures of the pulse exposure variant to increasing concentrations of IMD were performed over the next two months, allowing sufficient time for recovery between exposures, with the final re-exposure at 172 nM (FL Exp variant 1). The third method of FL IMD exposure involved continuous exposure to increasing concentrations of IMD, starting at the lowest previously determined IC_50_ of the strain (24 nM) and gradually increasing up to 115 nM (FL Exp variant 2).

The TX isolate was exposed continuously to 3.2 nM IMD for 24 days and also to a higher concentration (9.5 nM IMD) for a short period of time (11 days).

### Infection and treatment of ponies with Theileria equi

Four naïve mixed-breed ponies, 1–2 years of age, were experimentally infected with the USDA FL strain of *T. equi* via *Rhipicephalus microplus* tick transmission as a part of a separate research study, using previously published methods [42]. All ponies were confirmed positive for *T. equi* by nested PCR [4] and were initially treated with IMD (4 mg/kg, IM, q72 hrs for four doses) at seven months post-infection. Post-treatment infection status was determined using nested PCR and cELISA [43]. Because this initial treatment failed to clear *T. equi* in each of the four ponies, the same treatment regimen was repeated 18 months post-infection and the same follow-up evaluations performed. All experiments involving animals were carried out in accordance with the recommendations in the Guide for the Care and Use of Laboratory Animals of the National Institutes of Health and in conformance with the United States Department of Agriculture animal research guidelines, under a protocol approved by the Washington State University Institutional Animal Care and Use Committee (ASAF # 04163).

## Results

### Imidocarb dipropionate treatment of ponies experimentally infected with the USDA FL strain of Theileria equi

**Figure 1:**
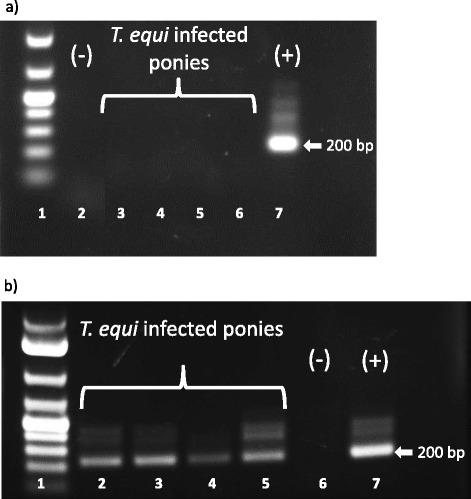
**Nested PCR detection of** ***T. equi*** **infection in ponies after failed IMD treatment. (a)** 2% agarose gel showing results from packed erythrocytes collected 16 days after the second round of IMD treatment. Lane 2: negative control erythrocytes from an uninfected horse (−). Lanes 3, 4, 5, 6: erythrocytes from the four IMD treated ponies. Lane 7: positive control erythrocytes from a known infected horse showing the 200 bp *T. equi ema-1* amplicon (+). **(b)** *T. equi ema-1* amplicons from packed erythrocytes collected 80 days after the second round of IMD treatment. Lanes 2, 3, 4, 5: erythrocytes from the four IMD treated ponies. Lane 6: negative control (−). Lane 7: positive control (+).

Sixteen days following the first round of IMD treatment (seven months post-infection), all four ponies appeared to be negative for *T. equi* based on nested PCR (Figure 1a). However, nested PCR was repeated at 17 months post-infection and all ponies were found to be positive. After the second round of IMD treatment at 18 months post-infection, the ponies again appeared negative for *T. equi* based on nested PCR performed 16 days post-treatment. However, re-evaluation 37 and 80 days (Figure 1b) after this second round of treatment (19–20 months post-infection) confirmed that all ponies again reverted to a positive state and that two rounds of IMD treatment failed to clear infection. Seropositivity was also confirmed at 14 and 20 months post-infection by cELISA (data not shown).

### *In vitro* susceptibility of Theileria equi to imidocarb propionate

**Figure 2:**
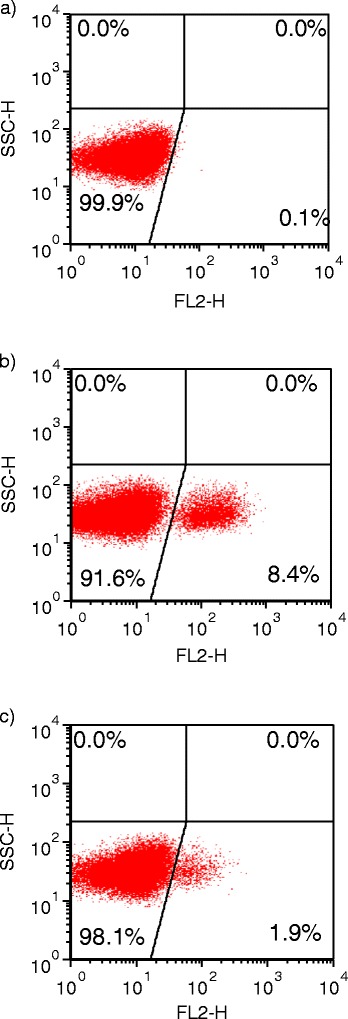
**Flow cytometric scatter plots depicting percent parasitized erythrocytes (PPE).** Representative samples from a 72 hour growth inhibition assay. Parasitized erythrocytes stained with hydroethidine were detected in the FL-2 channel, and are represented in the bottom right quadrant. **(a)** Normal uninfected erythrocytes stained with hydroethidine (negative control). **(b)** *T. equi-*infected erythrocytes incubated without imidocarb dipropionate (IMD; positive control). **(c)** *T. equi*-infected erythrocytes incubated with 2.76 μM IMD.

Development of the *in vitro* parasite growth inhibition assay using IMD for *T. equi* was successfully accomplished using the FL parasite strain, with evaluation of the PPE for each sample using flow cytometry (Figure 2). Regardless of the concentration of IMD, the PPE in all wells doubled or tripled over the first 24 hours. By 48 hours, the growth dynamics across IMD concentrations began to diverge, with parasite growth at higher concentrations leveling off and that at lower concentrations continuing to increase until reaching a maximum of 7-8% PPE at 72 hours. Overall, the final PPE across the range of drug concentrations reflected a dose-dependent effect of IMD, with an IC_50_ of 24 nM (Figure 2a). An IC_90_ could not be determined for this strain, because even at the maximum drug concentration (775 nM) the PPE still reached 1.2%, equivalent to 15.4% of the growth of untreated parasites. This indicated that the FL strain parasite population more than doubled from the starting PPE of 0.5% even at high IMD concentrations, reflecting a lack of complete growth inhibition.

**Figure 3:**
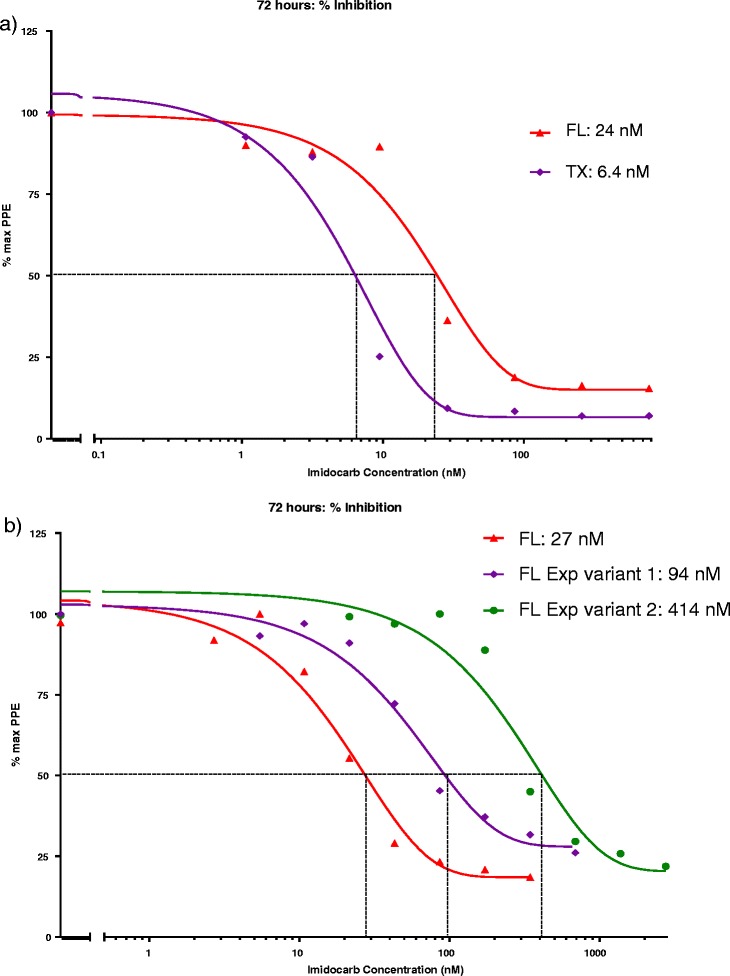
**Variable** ***in vitro*** **susceptibility to IMD among different** ***T. equi*** **isolates.** Each point on the nonlinear regression curve represents the mean percent of the maximum PPE determined in triplicate wells. IC_50_ is drug concentration resulting in 50% of the maximum PPE detected in wells not containing drug. **(a)** Susceptibility of the USDA FL strain (red) and the TX isolate (purple). **(b)** Susceptibility of FL Exp variant 1 (purple) and FL Exp variant 2 (green).

The TX isolate originating from the 2009 outbreak [4,6] was consistently more susceptible to IMD than the FL strain, with an IC_50_ of 6.4 nM; almost six-fold lower than FL (Figure 3a). In addition, an IC_90_ of 26 nM could be determined for the TX isolate, as it did not actively grow at higher drug concentrations, with PPE remaining at the starting level of 0.5%.

### Imidocarb dipropionate exposure of Theileria equi and the effect on IC_50_

Continuous exposure of the FL strain to increasing concentrations of IMD (up to 115 nM) increased the IC_50_ a maximum of 15-fold in FL Exp variant 2 to 414 nM (Figure 3b). Pulse exposure with IMD (up to 172 nM) increased the IC_50_ in FL Exp variant 1 to a lesser degree, approaching four-fold at 94 nM (Figure 3b). However, this effect could not be duplicated in the TX isolate. Despite repeated attempts to expose TX to IMD, the IC_50_ remained the same at approximately 6 nM. Exposure of TX to a higher concentration of IMD (9.5 nM) resulted in irrevocable decline in culture PPE and eventual death of all exposed parasites.

### Gatekeeper residue of Theileria equi calcium-dependent protein kinase

**Figure 4:**
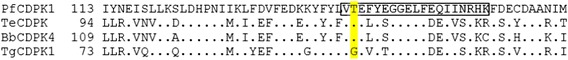
**Amino acid alignment through the ATP-binding domains of calcium dependent protein kinases.** Includes amino acid sequences from *P. falciparum* (Pf)*, T. equi* (Te), *B. bovis* (Bb)*, and T. gondii* (Tg). The ATP-binding region is boxed, and the gatekeeper residues are shaded.

Due to the variability in response to IMD, alternative drugs for treatment of *T. equi* were considered. The BKI compounds were of interest, and therefore evaluation of the *T. equi* transcriptome for an appropriate CDPK drug target was undertaken. Amino acid sequence comparisons of the BKI-binding CDPKs in *P. falciparum*, *B. bovis*, and *T. gondii* against the *T. equi* predicted transcriptome identified the same protein kinase domain-containing protein (GI: 510911326) as the putative CDPK ortholog in *T. equi*. Alignment of these four sequences revealed a threonine gatekeeper residue in *T. equi*, consistent with the small amino acid gatekeeper residues present in comparison organisms (Figure 4), all of which are susceptible to BKI compounds [33].

### In vitro susceptibility of Theileria equi to the bumped kinase inhibitor compound 1294

**Figure 5:**
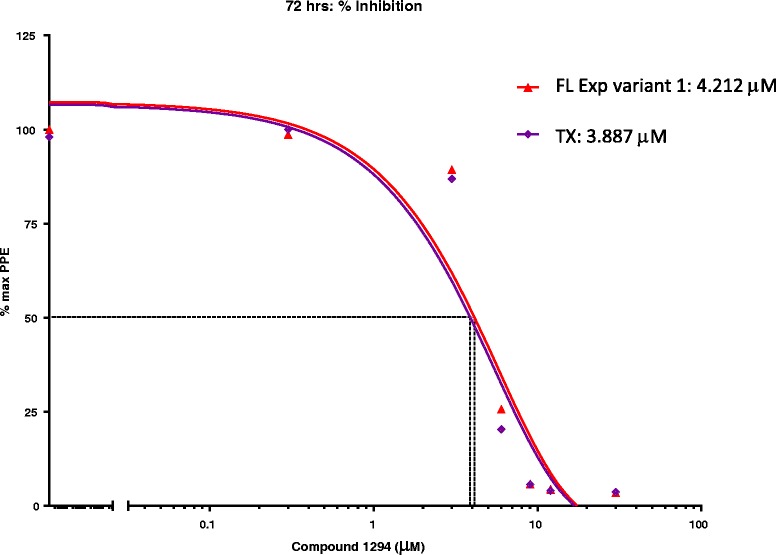
***In vitro*** **susceptibility of** ***T. equi*** **isolates to BKI compound 1294.** Each point on the nonlinear regression curve represents the mean percent of the maximum PPE determined in triplicate wells. IC_50_ is drug concentration resulting in 50% of the maximum PPE detected in wells not containing drug. Depicted results represent TX (purple) and FL Exp variant 1 (red).

For BKI 1294, the IC_50_ of the FL Exp variant 1 and the TX isolate were similar (4.212 μM and 3.887 μM respectively) (Figure 5). Active parasite growth of both isolates was completely inhibited at the highest drug concentrations, with PPE limited to the starting level of 0.3% (3.5-3.6% of the maximum PPE). Therefore, the IC_90_ was achieved for BKI 1294 for both FL Exp variant 1 and TX (11.502 μM and 10.964 μM, respectively).

## Discussion

Although the life cycle of *T. equi* in horses is biphasic, with an early pre-erythrocytic schizogony within leukocytes, this schizont stage is transient and does not appear to play a role in persistent infection of horses [42]. Instead, long term infection is perpetuated through infection and proliferation of merozoites in erythrocytes, making this merozoite stage most relevant for chemotherapeutic intervention. Therefore, erythrocyte cultures of merozoites were used in this study to evaluate *in vitro* susceptibility. The observed difference in IMD susceptibility between isolates, with the IMD IC_50_ of the FL strain almost six-fold higher than that of the TX isolate, was consistent with variation in efficacy that has been observed clinically. The overall success rate of IMD treatment for horses infected with the TX isolate during the 2009 outbreak was extremely high [4]. In contrast, the USDA FL strain has been used in multiple experimental settings to evaluate the efficacy of IMD *in vivo*, with infected horses often failing to clear FL following treatment with a protocol identical to that used in the TX outbreak [7,8]. This situation was also observed in the current study, with four FL-infected ponies failing to clear infection despite two rounds of IMD treatment at 4 mg/kg every 72 hours for 4 doses per round. Interestingly, the ponies appeared negative immediately after treatment, with parasite growth suppressed by IMD to the point that it could not initially be detected even with the extremely sensitive nested PCR method, which has been shown to detect a level of parasitemia of 0.000006% [44].

Previous research has demonstrated the presence of *T. equi* in the spleen of asymptomatic horses at times where it was undetectable in the peripheral blood via multiplex PCR [2]. Additionally, it is possible that the level of parasitemia was simply lower than the detectable limit, and that the small number of parasites surviving drug treatment was still sufficient to allow continued infection after a period of parasite recovery. The lack of complete *in vitro* inhibition of FL is consistent with the observed failure of IMD to clear the FL strain in the infected ponies described in this study, as incomplete parasite inhibition led to a resurgence of parasitemia *in vivo*. This also raises further concern about the potential for development of resistance in these IMD-exposed surviving parasites.

The pharmacokinetics of IMD administered intramuscularly in horses demonstrates a maximum plasma concentration of 0.2 μg/mL (574 nM) at a dose of 2.4 mg/mL [39]. Although this concentration greatly exceeds the IC_50_ of 24 nM observed for the FL strain in the present study, this plasma concentration is maintained for only 2 hours *in vivo* and drops to undetectable levels (less than 0.0125 μg/mL, or 36 nM) after 12 hours [39]. In contrast, the parasites in the present *in vitro* assay were exposed to IMD continuously at the tested concentrations for 72 hours, and in the case of FL, actively grew despite exposure to high concentrations of drug.

It has been postulated that a reservoir effect exists for IMD *in vivo*, as the drug is deposited in the liver and kidneys [45-48] as well as muscle in cattle [48] during the initial distribution phase. Trace amounts have been detected in the plasma of sheep up to four weeks after treatment [47]. This likely accounts for the overall efficacy of IMD for certain hemoprotozoan parasites, but makes it difficult to compare *in vitro* IC_50_ results with circulating IMD concentrations to determine if adequate drug concentrations are reached in the plasma. Additionally, the minimum detectable concentration of 0.0125 μg/mL (36 nM) for the HPLC assay utilized in the previous study [39] exceeded the IC_50_ of IMD for both FL and TX observed in the current study. Therefore, it is possible that the plasma concentration remains higher than the IC_50_ for a period longer than that suggested by the previous pharmacokinetics study. The 2.4 mg/kg dose used in that study was less than the 4 mg/kg dose currently recommended for clearance of *T. equi* [4,7]. Although the higher dose would be expected to achieve higher plasma concentrations, the fact that treatment failures occur at the higher dose indicates that IMD plasma concentrations above the IC_50_ are not always adequate for elimination of *T. equi in vivo*_._

We observed that the IC_50_ for the FL strain could be further increased *in vitro* by exposure to IMD, as has been shown previously *in vitro* for *B. bovis* [22]. This finding suggests that resistance could emerge in natural parasite populations with exposure to IMD when animals are treated, particularly if complete inhibition of parasite growth cannot be achieved. As previously mentioned, trace amounts of IMD can be found in the plasma of sheep up to four weeks following treatment with IMD [47], which could result in prolonged drug selection pressure if the same occurs in horses. Although horses are not currently treated routinely in the United States, treatment occurs much more frequently in endemic countries. In these endemic countries the goal of therapy is to treat clinical disease rather than eliminate the parasite entirely from the host [49]. Therefore, treated horses can remain chronically infected and retain a population of parasites that was exposed to IMD yet was not killed by the drug. The possibility of developing IMD resistance is concerning, as there are very few options currently available for treatment of *T. equi*. In contrast to the observations with the FL strain however, IMD susceptibility in the TX isolate was not altered despite repeated IMD exposure. Thus, the capacity for drug exposure-associated changes in *in vitro* IMD susceptibility varies among *T. equi* strains. The underlying mechanism of IMD resistance among different *T. equi* strains is a focus of ongoing investigation.

The BKIs represent a novel class of compounds with potential as safe and effective treatment alternatives for *T. equi* infection, particularly for horses infected with a strain that is less susceptible to IMD. Compound 1294 is safe *in vivo* in rodents and effective against *T. gondii* [30], *C. parvum* [32], *P. falciparum* [29] and *B. bovis* [33]. Importantly, it is nontoxic in mice following twice daily oral administration of 100 mg/kg for five days [29]. It has 91% oral bioavailability in rats and is likely cleared by hepatic metabolism [29]. In the current study, this compound demonstrated equal and substantial efficacy *in vitro* against both the TX isolate and the FL Exp variant 1, which had an almost 15-fold difference in IMD susceptibility. Importantly, BKI 1294 resulted in complete parasite growth inhibition for both isolates, consistent with its novel mechanism of action. Although the IC_50_ of BKI 1294 was higher than that of IMD *in vitro*, its apicomplexan-selective target and its favorable pharmacokinetics and safety in other mammals [28-30] suggest that it could be useful for eliminating *T. equi* infection in horses. Further investigation of this class of compounds for this purpose is a focus of ongoing research.

Flow cytometry has been used to evaluate parasite infection of erythrocytes for multiple hemoprotozoan species, including *B. bovis* [50], *Anaplasma marginale* [51], *B. gibsoni* [52], *B. canis* [53], and *P. berghei* [54]*.* This technique relies on staining of infected erythrocyte populations with hydroethidine, which is taken up by live parasites within intact erythrocytes and converted to the fluorochrome ethidium bromide. Ethidium bromide intercalates into the DNA of viable parasites and can be utilized to differentiate infected erythrocytes from those that are uninfected or that contain non-viable parasites using flow cytometry [50]. It is therefore particularly relevant for assessment of drug susceptibility as only viable parasites are identified, in comparison to the traditional method of evaluating PPE by blood smear in which parasite viability cannot be determined. In this study, we were able to successfully apply this technique for *T. equi*-infected erythrocytes for the first time to evaluate parasite growth in drug susceptibility assays.

## Conclusions

This study demonstrated variation in IMD susceptibility for the first time *in vitro* between two *T. equi* strains, with decreased susceptibility and incomplete parasite inhibition for the USDA FL strain. This finding was consistent with the inability of IMD to clear the same strain *in vivo* in four experimentally infected ponies, despite two rounds of treatment. Importantly, the emergence of IMD resistance during exposure to the drug was demonstrated *in vitro.* In contrast, no such *in vitro* variation in susceptibility between strains was observed for BKI compound 1294. This novel class of compounds may represent an effective alternative for the treatment of resistant *T. equi* infections in horses.

## Abbreviations

BKI: Bumped kinase inhibitor
CDPK: Calcium-dependent protein kinase
FL: USDA Florida strain of *T. equi*
FL Exp variant 1: *T. equi* variant pulse exposed to IMD
FL Exp variant 2: *T. equi* variant continuously exposed to IMD
IC_50_: 50% inhibitory concentration
IC_90_: 90% inhibitory concentration
IMD: Imidocarb dipropionate
PPE: Percent parasitized erythrocytes
TX: novel *T. equi* isolate originating from Texas
